# Sex-based differences in transition readiness and health-related quality of life during the healthcare transition of youth with special health care needs

**DOI:** 10.1016/j.clinsp.2026.101030

**Published:** 2026-07-08

**Authors:** Ana Paula S. Ferrer, Clara R. Doria, Claudia B. Fonseca, Darusa C. Souza, Flavia C. Yarshell, Pedro Henrique P. Sampaio, Adriana M. Elias, Lucia Maria A. Campos, Andreia Watanabe, Caroline Gouveia Buff Passone, Louise Cominato, Maria Teresa Terreri, Artur F. Delgado, Clovis Artur Silva

**Affiliations:** a Instituto da Criança e Adolescente, Hospital das Clínicas da Faculdade de Medicina da Universidade de São Paulo (HCFMUSP), São Paulo, SP, Brazil; b Pediatric Rheumatology Service, Universidade Federal de São Paulo, São Paulo, Brazil

Children and Youth with Special Health Care Needs (CYSHCN) include various chronic conditions characterized by varying clinical severities and functional limitations.^1^ The prevalence of CYSHCN ranged from 15% to 25% of the pediatric population,^2^^,^^3^ with youth aged 12 to 17 years (YSHCN) representing over 40% portion of this cohort.^2^ Recent studies showed differences comorbidities between males and females’ YSHCN.^4^^,^^5^ Boys are more likely than girls to have higher rates of medications, specialized therapy requirements, and behavioral or educational challenges.^5^

In addition, most adolescents with chronic conditions now survive into adulthood, demanding consistent long-term follow-up. Therefore, healthcare transition defined as a planned movement of adolescents and young adults with chronic conditions from adolescent-centered to adult-oriented systems,^6^ becomes a critical challenge, particularly for YSHCN populations.^7^

YSHCN often have complex and rare chronic conditions necessitating specialized care and more support than their peers.^8^^,^^9^ Despite the importance of the healthcare transition process, there is no report to evaluate concomitantly transition readiness and Health-Related Quality of Life (HRQL) instruments between male and female YSHCN population across diverse pediatric subspecialties. Thus, the objective of the present study is to assess demographic data, National Survey of Children’s Health (NSCH) criteria of YSHCN, and transition readiness and HRQL parameters in male versus female YSHCN during transition process from pediatric to adult healthcare.

This is a cross-sectional prospective approved by the Ethics Committee of our tertiary hospital (CAEE 84,030,924.4.0000.0068). Informed assent and informed consent were obtained from all YSHCN and their parents/guardians, respectively. From March 2025 to April 2026, we evaluated 220 consecutive YSHCN from 13 outpatient clinics at one tertiary and university hospital. All our YSHCN are in healthcare transition process from pediatric to adult-oriented services. Two were excluded due to incomplete data, and the remaining 218 were systematically evaluated demographic data, NSCH criteria of YSHCN,^10^ Transition Readiness Assessment Questionnaire (TRAQ)[11] and HRQL[12] parameters, medications and pediatric specialties.

TRAQ is a self-administered tool, validated in Brazilian Portuguese language and contains 18 questions divided into four domains: “taking medication”, “going to appointments”, “tracking health problems”, and “daily activities”. Estimated scale scores were calculated using the average score of items in each domain and resulting in an overall scale score also varying from 1 to 5, with a higher score indicating greater readiness for transition.^11^

HRQL was carried out using the Pediatric Quality of Life Inventory 4.0 (PedsQL4.0). The PedsQL4.0 is a generic, multidimensional, self-administered toll, validated in Brazilian Portuguese language for adolescents. This instrument contains 23 items divided into four domains: physical capacity, emotional issues, social aspects, and school/work activities, assessed in the last month of study entry. Psychosocial health included emotional, social, and school functioning domain scores. Higher scores indicated better HRQL.^12^

Data were captured by Research Electronic Data Capture (REDCap). Results were presented as median (interquartile range) for continuous variables, and number (%) for categorical variables. For categorical variables, differences were assessed using Pearson's chi-square test or Fisher exact test, as appropriated. The Mann-Whitney *U test* was used to compare medians (interquartile range) with non-normal distribution. Correlations between current age, number of residents in the house, number of rooms in the house, and TRAQ and PedsQLTM 4.0 scores were analyzed by Spearman's rank correlation coefficient. The significance level was set at 5% (p < 0.05).

Table 1 includes comparison between demographic data, NSCH criteria of YSHCN, transition readiness and HRQL parameters in male versus female YSHCN during the transition from pediatric to adult healthcare.

**Table 1:** Comparison between demographic data, NSCH criteria of Youth with Special Health Care Needs (YSHCN), transition readiness and Health-Related Quality of Life (HRQL) parameters, medications and pediatric specialties in male versus female YSHCN during the transition process from adolescent to adult healthcare.

Variables n (%)	Male YSHCN (n = 105)	Female YSHCN (n = 113)	p
Demographic data			
Current age, years	16.3 (15.6‒17.25)	16.4 (15.3‒17.5)	0.746
Race/Ethnicity			0.620
Caucasian	48 (45.7)	50 (44.2)	‒
Brown	40 (38.0)	41 (36.3)	‒
Black	14 (13.3)	19 (16.8)	‒
Indigenous	1 (1.0)	3 (2.7)	‒
Yellow	1 (1.0)	0 (0.0)	‒
Others	1 (1.0)	0 (0.0)	‒
Number of minimum wages/family			0.073
Until 2 minimum wages	39 (37.1)	57 (50.4)	‒
2 to 4 minimum wages	40 (38.1)	29 (25.7)	‒
4 to 10 wages	22 (21.0)	26 (23.0)	‒
Over 10 wages	4 (3.8)	1 (0.9)	‒
Educational data			0.238
Incomplete literacy	1 (1.0)	0 (0.0)	
Incomplete elementary and middle school	17 (16.2)	24 (21.2)	
Complete elementary and middle school	2 (1.9)	1 (0.9)	
Incomplete high school	80 (76.2)	76 (67.3)	
Complete high school	2 (1.9)	9 (8.0)	
Incomplete college/university	3 (2.9)	2 (1.8)	
Complete college/university	0 (0.0)	1 (0.9)	
Number of rooms in the house	5.0 (5.0‒7.0)	6.0 (5.0‒7.0)	0.473
Number of residents in the house	4.0 (3.0‒4.0)	4.0 (3.0‒4.0)	0.840
NSCH criteria of YSHCN (1, 2 and at least one of 3)	105 (100.0)	113 (100.0)	1.000
1. Medical or other health condition	105 (100.0)	113 (100.0)	1.000
2. Duration of medical or other health condition ≥ 12 months	105 (100.0)	113 (100.0)	1.000
3. At least one specific consequence experienced:	105 (100.0)	113 (100.0)	1.000
a) Need or prescription medication(s) use	103 (98.1)	112 (99.1)	0.610
b) Elevated need or medical care use, mental health, or education services	103 (98.1)	113 (100.0)	0.231
c) Functional limitation(s) limiting daily activity	15 (14.3)	17 (15.0)	1.000
d) Need for specialized therapies (physical, occupational, or speech therapy)	64 (61.0)	73 (64.6)	0.674
e) Developmental or behavioral problems for which treatment or counseling is needed	17 (16.2)	42 (37.2)	<0.001
TRAQ score			
Total TRAQ	3.6 (3.3‒3.9)	3.8 (3.5‒4.3)	0.001
Taking medication (score 1‒5)	4.0 (3.2‒4.2)	4.0 (3.8‒4.8)	0.006
Going to appointments (score 1‒5)	3.1 (2.7‒3.6)	3.3 (2.9‒3.9)	0.009
Tracking health problems (score 1‒5)	3.1 (2.7‒3.6)	3.3 (2.9‒3.9)	0.009
Daily activities (score 1‒5)	4.7 (4.0‒5.0)	4.7 (4.0‒5.0)	0.043
HRQL (PedsQL 4.0) score			
PedsQL 4.0 total score (0‒100)	75.0 (64.1‒83.7)	64.1 (53.3‒75.0)	<0.001
Physical (0‒100)	87.5 (75.0‒93.8)	68.8 (59.4‒84.4)	<0.001
Physicosocial (0‒100)	70.0 (58.3‒80.0)	60.0 (48.3‒71.7)	<0.001
Social (0‒100)	80.0 (70.0‒95.0)	75.0 (60.0‒90.0)	0.046
Emotional (0–100)	65.0 (55.0‒75.0)	50.0 (35.0‒65.0)	<0.001
School/work (0‒100)	60.0 (50.0‒70.0)	55.0 (43.8‒65.0)	0.033
Medications			
Number of medications per patient	5.0 (3.0‒7.0)	5.0 (3.0‒7.0)	0.938
Polypharmacy ≥5 medications	60 (57.1)	64 (56.6)	1.000
Polypharmacy ≥10 medications	11 (10.5)	8 (7.1)	0.473
Pediatric specialties			
≥3 specialties per patient	33 (31.4)	42 (37.2)	0.395
1. Cardiology	0 (0.0)	1 (0.9)	1.000
2. Endocrinology	8 (7.6)	12 (10.6)	0.489
3. Gastroenterology/Nutrology	13 (12.4)	9 (8.0)	0.369
4. Genetics	1 (1.0)	0 (0.0)	0.482
5. Hematology	12 (11.4)	12 (10.6)	1.000
6. Hepatology and Liver Transplantation	11 (10.5)	20 (17.7)	0.174
7. Immunology and Allergy	5 (4.8)	8 (7.1)	0.573
8. Nephrology and Renal Transplantation	22 (21.0)	17 (15.0)	0.291
9. Neurology	5 (4.8)	6 (5.3)	1.000
10. Pediatric surgery	1 (1.0)	1 (0.9)	1.000
11. Pneumology	15 (14.3)	6 (5.3)	0.037
12. Psychiatry	3 (2.9)	2 (1.8)	0.674
13. Rheumatology	18 (17.1)	36 (31.9)	0.013

Results are presented in n (%) and median (interquartile range). NSCH, National Survey of Children’s Health; TRAQ, Transition Readiness Assessment Questionaire; PedsQL 4.0, Pediatric Quality of Life Inventory 4.0 scores.

All 218 (100%) subjects fulfilled NSCH criteria of YSHCN.^10^ The 10 most important pediatric chronic conditions are: childhood-onset systemic lupus erythematosus (n = 25, 11.2%), kidney transplantation (n = 28, 12.6%), autoimmune hepatitis (n = 19, 8.5%), sickle cell disease (n = 16, 7.2%), cystic fibrosis (n = 13, 5.8%), Crohn's disease (n = 13, 5.8%), juvenile idiopathic arthritis (n = 8, 3.6%), ulcerative colitis (n = 8, 3.6%), severe obesity/bariatric surgery (n = 7, 3.1%) and diabetes mellitus (n = 6, 2.7%).

The median of current age, number of rooms in the house and number of residents in the house were similar in male and female YSHCN (p > 0.05), as well as the frequencies of race/ethnicity and number of minimum wages/family (p > 0.05).

According to the different NSCH criteria for YSHCN, the prevalence of developmental or behavioral problems requiring treatment or counseling was significantly lower in male than female YSHCN (16.2%vs. 37.2%, p < 0.001). The median of total TRAQ score (p = 0.001) and the median of four TRAQ domains: taking medication (p = 0.006), going to appointments (p = 0.009), tracking health problems (p = 0.009) and daily activities (p = 0.043) were significantly reduced in male that female YSHCN. Whereas the median of PedsQL 4.0 total score (p < 0.001), physical (p < 0.001), physicosocial (p < 0.001), social (p = 0.046), emotional (p < 0.001), and school/work (p = 0.033) were significantly higher in male that female YSHCN (Table 1).

Regarding specialty care, male YSHCN were significantly more likely to be followed by Pneumology than female (14.3%vs. 5.3%, p = 0.037), and significantly lower rates of Rheumatology follow-up in the former group (17.1%vs. 31.9%, p = 0.013). No differences were evidenced in the number of medications per patient and polypharmacy in both groups (p > 0.05) (Table 1).

Positive Spearman's rank correlation coefficients were observed between current age and mean total TRAQ in all YSHCN (n = 218, *r* = +0.186, p = 0.006), and in female YSHCN (n = 113, *r* = +0.211, p = 0.025). In contrast, negative Spearman's rank correlation coefficients were observed between number of residents in the house and mean total TRAQ in all YSHCN (n = 218, *r* = **−**0.14, p = 0.039), and in male YSHCN (n = 105, *r* = **−**0.212, p = 0.03).

To our knowledge, the present study was the first to evaluate adolescents with pediatric chronic conditions using NSCH criteria of YSHCN during the transition process, particularly assessing TRAQ and HRQL scores. We showed that male YSHCN demonstrated lower transition readiness across multiple domains of the TRAQ, specifically in drug management, appointment attendance, and tracking health issues.

Interestingly, although males YSHCN were less prepared for transition readiness, they reported higher HRQL scores in all PedsQL4.0 domains. This discrepancy may suggest that females YSHCN may perceive the burden of their chronic conditions more importantly, perhaps due to a higher level of engagement and responsibility for their own care, which in turn increases their readiness. Female YSHCN also presented developmental or behavioral vulnerabilities where counseling could provide meaningful support.

In conclusion, the present report identified a sex-based disparities in YSHCN. Despite reporting better HRQL, male patients showed delay in transition readiness and female revealed more behavioral challenges. These findings highlighted that healthcare transition programs should implement sex-tailored strategies to address the distinct self-management deficits and psychosocial requirements to minimize the risk of adverse health outcomes during young adulthood.

## Declaration of generative AI and AI-assisted technologies in the manuscript preparation process

During the preparation of this work the author(s) used Goggle Gemini exclusively to improve the English phrasing and clarity of the manuscript. After using this tool/service, the author(s) reviewed and edited the content as needed and take(s) full responsibility for the content of the published article.

## Data availability

The datasets generated and/or analyzed during the current study are available from the corresponding author upon reasonable request.

## Declaration of competing interest

The authors declare no conflicts of interest.
